# Effects of high resistance muscle training on corticospinal output during motor fatigue assessed by transcranial magnetic stimulation

**DOI:** 10.3389/fphys.2023.1125974

**Published:** 2023-03-10

**Authors:** Anelia Dietmann, Marisa Blanquet, Kai Michael Rösler, Olivier Scheidegger

**Affiliations:** ^1^ Department of Neurology, Inselspital, Bern University Hospital, University of Bern, Switzerland, Bern, Switzerland; ^2^ Neurozentrum Basel, Bellevue Medical Group, Basel, Switzerland; ^3^ Institute for Diagnostic and Interventional Neuroradiology, Inselspital, Bern University Hospital, University of Bern, Bern, Switzerland

**Keywords:** fatigue, exercise, TMS, motor-evoked potentials, healthy, resistance training

## Abstract

**Introduction:** Central fatigue refers to a reduced drive of motor cortical output during exercise, and performance can be enhanced after training. However, the effects of training on central fatigue remain unclear. Changes in cortical output can be addressed non-invasively using transcranial magnetic stimulation (TMS). The aim of the study was to compare responses to TMS during a fatiguing exercise before and after a 3 weeks lasting resistance training, in healthy subjects.

**Methods:** The triple stimulation technique (TST) was used to quantify a central conduction index (CCI = amplitude ratio of central conduction response and peripheral nerve response) to the abductor digiti minimi muscle (ADM) in 15 subjects. The training consisted of repetitive isometric maximal voluntary contractions (MVC) of ADM for 2 min twice a day. Before and after this training, TST recordings were obtained every 15 s during an 2 min exercise of MVC of the ADM, where subjects performed repetitive contractions of the ADM, and repeatedly during a recovery period of 7 min.

**Results:** There was a consistent decrease of force to approximately 40% of MVC in all experiments and in all subjects, both before and after training. In all subjects, CCI decreased during exercise. While before training, theCCI decreased to 49% (SD 23.7%) after 2 min of exercise, it decreased after training onlyto 79% (SD 26.4%) after exercise (*p* < 0.01).

**Discussion:** The training regimen increased the proportion of target motor units that could be activated by TMS during a fatiguing exercise. The results point to a reduced intracortical inhibition, which may be a transient physiological response to facilitate the motor task. Possible underlying mechanisms at spinal and supraspinal sites are discussed.

## 1 Introduction

Muscle fatigue is specified as an exercise-induced decrease of the force generated by a muscle (Edwards et al., 1977). It is caused by alterations of structures involved in movement generation from the brain to the muscle cells. A progressive failure to drive motor neurons (MNs) during exercise has been termed central fatigue (Gandevia, 2001). It is associated with changes of excitability of the involved neural cells within the central nervous system (CNS) at spinal and supraspinal sites. Therefore, the size of motor evoked potentials (MEPs) elicited by transcranial magnetic stimulation (TMS) of the brain is altered after fatiguing contractions of muscles (Brasil-Neto et al., 1993; McKay et al., 1995; Liepert et al., 1996; Samii et al., 1997; Gandevia et al., 1999; Pitcher and Miles, 2002; Andersen et al., 2003; Rösler et al., 2009).

In a previous study on TMS-induced responses during muscular fatigue, we used a novel technique of TMS during a fatiguing exercise and demonstrated a dramatic decrease of the MEPs that could be evoked, indicating a decreased responsiveness of the MNs to synaptic input (Rösler et al., 2009). This decrease was paralleled by a significant drop of muscle force. We suggested an important supraspinal contribution mediated by intracortical inhibition and voluntary drive, while spinal mechanisms seem to have little effect on the MEP reduction. While the decrease of muscle force was fairly similar in all subjects, a remarkable inter-individual variability in the behavior of the TMS evoked responses was observed. A possible cause of these inter-individual differences were differences of the individual state of muscle training among the subjects. If this was the case, then muscular training could influence the reduced MN activation during a fatiguing exercise.

Muscular strength training is known to lead to adaptations not only within the muscle itself, but also within the CNS, as summarized in a review by Kidgell and Pearce (2011). Central nervous system adaptations to training have been inferred by the observation that at the beginning of a strength training program, muscle force increases without concomitant increase of muscle cross-sectional area (Lüthi et al., 1986; Griffin and Cafarelli, 2005). Hence, at the beginning of a training regimen, when structural adaptations within the muscle tissue have not yet occurred, CNS adaptations may precede and serve to increase the efficiency of a muscle generating its force output. Strength training does not increase the size of MEPs when the measurements are done in rested, unfatigued muscles at rest (Carroll et al., 2002; Jensen et al., 2005; Duchateau et al., 2006). The present experiments were done to test the hypothesis that a 3 weeks resistance training program could induce CNS adaptations which in turn would alter the MNs’ susceptibility to TMS during fatigue. In contrast to previous studies, we quantified the size of MEPs using the triple stimulation technique (TST), which increases the sensitivity for detection of small changes (Magistris et al., 1998; Humm et al., 2004). Moreover, we measured the MEPs not only in rested muscles but also during a fatiguing exercise, since the size of MEPs changes dramatically in fatigued muscles.

## 2 Materials and methods

### 2.1 Subjects

Fifteen healthy subjects, 8 women and 7 men, aged 22–35 years (mean age 25 years) participated in this study and underwent approx. 3 weeks of training of fifth finger abduction. Measurements were performed before and after this training regimen, as outlined below. None of the subjects had a history of previous neurological disorders or a contraindication to TMS (e.g., implanted metal in the eye or brain, cardiac pacemaker). The study was approved by the local ethics committee and all subjects gave their written informed consent.

### 2.2 Electrophysiological and mechanical recordings

Responses were recorded from the abductor digiti minimi muscle (ADM) of the non-dominant hand in 15 subjects (14 recordings on the left). The ADM was used for recordings because in this muscle there is only little volume conduction from neighboring muscles (interfering with the recordings) (Magistris et al., 1998; Humm et al., 2004), and because a fatigue protocol had previously been established using this muscle (Rösler et al., 2009).

Compound muscle action potentials (CMAPs) were recorded using a muscle-belly tendon montage with silver surface electrodes (diameter 0.8 cm). A ground electrode was taped to the wrist. For the recordings, a VIKING SELECT EMG apparatus was used (Nicolet Biomedical, Madison, Wisconsin, United States). Bandpass filtering was 2 Hz–10 kHz.

To measure the isometric voluntary contraction force of fifth finger abduction, the little finger was placed on a lever attached to a force transducer (Sensotec Inc., Columbus, Ohio, United States). The lever was parallel to the little finger when the hand is in neutral position, hence the joint angle was kept throughout the experiment at 0°. A platform in front of subjects held the force transducer and the lever. The subjects sat on a chair, their left forearm and hand attached with Velcro straps to the platform. This construction limited forearm and hand movements other than the abduction of digit V. A DC-amplifier was used to amplify the output signal of the force transducer (Sedia, Givisiez, Switzerland). It was then sampled at 4 kHz by AD converter (MacLab, ADInstruments Pty Ltd., Castle Hill, NSW, Australia) connected to a personal computer (Macintosh, Apple Computer Inc., Cupertino, California, United States) and stored for later off line analysis. During the experiments, the force signal was displayed on the computer screen for visual feedback (Arányi et al., 1998; Rösler et al., 2002; Rösler et al., 2009). The force could only be measured in percent of the maximal voluntary isometric contraction force (MVC) due to software restrictions.

### 2.3 Peripheral nerve stimulation

The ulnar nerve was stimulated at the wrist according to standard methods (Chen et al., 2016). The brachial plexus was stimulated at Erb’s point, using a pseudo-monopolar electrode montage, as described previously (Roth and Magistris, 1989; Magistris et al., 1998; Magistris et al., 1999). A small cathode electrode was taped over Erb’s point (diameter 1 cm) and a large remote anode electrode (surface area, 30 cm^2^) was attached to the internal region of the suprascapular fossa.

### 2.4 Transcranial magnetic brain stimulation

Motor evoked potentials were obtained using a MAGSTIM 200 stimulator (Magstim Company Ltd., Spring-Gardens, Whitland, United Kingdom) connected to a circular 90 mm hand-held coil. The center of the coil was at the vertex or slightly lateral toward the stimulated hemisphere. Face “A” (face visible) was used for stimulation of the left hemisphere (in one subject; current in counterclockwise direction) and face “B” for stimulation of the right hemisphere (current in clockwise direction). The coil was positioned in the area yielding the lowest threshold of the MEP. All further magnetic transcranial stimuli were applied at this position to avoid MEP changes by altered coil position (Magistris et al., 1998; Rösler et al., 2008). The stimulus intensity yielding a response of the relaxed target muscle in 50% of 8 or 10 trials at a gain of 100*μV*/*division* was defined as being the resting motor threshold (Rothwell et al., 1999). During the experiment, the intensity of the magnetic stimulus was chosen such that maximal (or near maximal) responses were obtained. All transcranial stimulation were given using the triple stimulation technique (TST) as outlined below.

### 2.5 Triple stimulation technique

**FIGURE 1:**
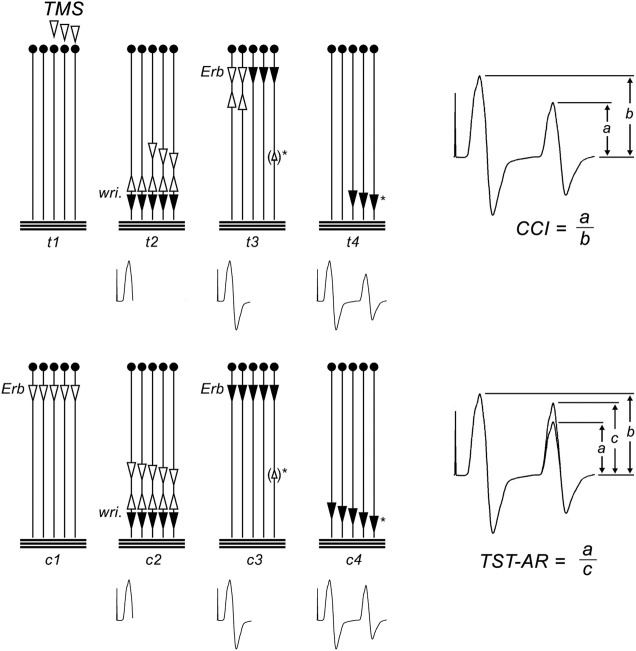
Triple stimulation technique (TST) principle. The motor tract is simplified to 5 spinal motor neurons (MNs); horizontal lines represent the muscle fibers of the target abductor digiti minimi (ADM) muscle. Black arrows represent action potentials (APs) that cause a trace deflection, open arrows those that do not. Below, the trace recording is given at each time point. **Upper panel:** TST test recording and calculation of the central conduction index (CCI); (t1) submaximal TMS excites 3 of 5 MNs. (t2) On 3/5 MNs, TMS induced APs descend. The 3 APs are not synchronous. After a delay, a supramaximal stimulus is applied to the ulnar nerve at the wrist (wri). It gives rise to a first negative deflection of the recording trace. The antidromic APs collide with the descending APs on MNs 3, 4, and 5. The AP on MNs 1 and 2 continue to ascend. (t3) After a second delay a supramaximal stimulus is given to the brachial plexus at Erb’s point. On MNs 1 and 2, the descending APs collides with the ascending APs. On neurons 3–5, no collision occurs, and APs descend. During their descent, a minor degree of desynchronization occurs, as typical for peripheral nerves. In some subjects, ephaptic muscle-nerve backfiring will occur (small open arrow in brackets)*, causing an AP to ascend on neuron 5. (t4) APs on MNs 3–5 cause a second negative deflection of the recording trace. Note that MNs 3–4 were those initially excited by the transcranial stimulus. The response is smaller than the first deflection because it results only fom 3 of the 5 MNs. It is additionally reduced because some desynchronisation of APs will occur during conduction from Erb’s point to the wrist, and because backfiring causes ascending APs (△)* which will collide with some of the descending APs. The CCI is calculated as the ratio of second and first deflection (CCI = a/b; right panel). **Lower panel:** TST control recording and calculation of the TST amplitude ratio (TST-AR); (c1) a maximal stimulus is given at Erb’s point. (c2) After a delay, a supramaximal stimulus at the wrist causes a first negative deflection of the TST control trace. (c3) After a second delay, a supramaximal stimulus is applied to Erb’s point, evoking APs on all MNs. During their descent, a minor degree of desynchronization occurs, matching (and calibrating) the desynchonisation that occurred during the TST test procedure. If muscle-nerve backfiring occurs ()*, it will also match that during the TST test procedure. (c4) A response from the 5 MNs is recorded as the second deflection of the TST control trace. It will be smaller than the first deflection, caused by peripheral desynchronisation and ephaptic backfiring. The test response is quantified as the ratio of TST test: TST control responses, thereby eliminating influences by peripheral desynchronisation and backfiring (TST amplitude ratio = a/c; right panel). Figure adapted from Z’Graggen et al. (2008).

Conventional TMS evokes muscle responses which vary in size and configuration from one stimulus to the next. This variability is caused by different degrees of desynchronization of the TMS induced MN discharges and by varying numbers of repetitively discharging units. In the present study, we used the TST to eliminate these effects, thus allowing for a quantification of the target MNs driven to discharge, in percent of the MN pool supplying the target muscle. The TST is a collision method described in detail previously (Magistris et al., 1998; Magistris et al., 1999; Rösler et al., 2002). It uses a sequence of three stimuli–a magnetic transcranial brain stimulus, and a supramaximal electrical stimulus of the ulnar nerve (wrist) and the brachial plexus (Erb’s point). These stimuli are suitably timed to yield a TST test curve, recorded from the target ADM. The succession of events that occur during the TST procedure are summarized in Figure 1. As a result of the 3 stimuli, the recording trace includes an M-response to supramaximal ulnar nerve stimulation (i.e., the first deflection of the TST trace; Figure 1, upper panel). It then includes a second deflection, corresponding in size to the number of MNs that were driven to discharge by TMS (Figure 1, upper panel). In the original TST protocol (Magistris et al., 1999), we quantified this second deflection by comparing it with a TST control curve, obtained by three successive supramaximal electrical stimuli given to the brachial plexus, the ulnar nerve, and the brachial plexus again (Figure 1, lower panel). The amplitude and area ratio of TST test: TST control (termed “TST amplitude ratio”) quantified the proportion of the target muscles MNs which were brought to discharge by TMS (Figure 1, lower panel) (Magistris et al., 1998). The timing during the fatigue experiments in the present study did not permit recording of TST control curves. Therefore, quantification of the responses was modified, and the second deflection was directly compared to the first deflection (Figure 1, upper panel). The resulting ratio was termed “central conduction index” (CCI, for a detailed discussion see (Magistris et al., 1998; Rösler et al., 2009). In a previous study we confirmed that the difference between CCI and the TST test: TST control ratio remained unchanged during the fatigue protocol applied here (Rösler et al., 2009).

### 2.6 Experimental protocol

Subjects were examined twice, in a session before and after training. Maximal voluntary contraction force was determined at the beginning of each of the two sessions, and was referenced throughout the corresponding experiment. All measurements started with the recording of 3 TST test curves and 1 TST control curve, while the subjects performed a contraction of 20% MVC of the respective session to facilitate the responses (Rösler et al., 2002). The stimulus intensity was 100% of stimulator output such that the TST amplitude ratio was close to 100% (i.e., that nearly 100% of MNs were driven to discharge). After the initial measurements, the subjects performed a fatiguing exercise of 2 min duration, by repetitive maximal abductions of the little finger, of 1 s duration each, at a rate of 0.5 Hz. During these 2 min, the subjects were asked every 15 s to perform a contraction at 20% of the initial MVC, and a TST test was performed. Following the exercise, the TST test was repeatedly performed during short contractions of 20% of the initial MVC, after 15, 30, 45, 60, 120, 240, 480, and 680 s, to assess changes during recovery. The maximal voluntary force was determined at each of these time points. Four hundred and 20 seconds after the end of the fatiguing exercise, a TST control curve war recorded. After 680 s, a TST control curve and a CMAP from peripheral nerve stimulation at the wrist was obtained.

### 2.7 Training protocol

The training started the day after the first measurement and terminated the day before the final measurement. Subjects were asked to perform a training of 3 weeks duration. To train the ADM, subjects were asked to perform repetitive isometric abductions of the little finger against resistance, with maximal force, at a frequency of about 0.5 Hz (similar to the fatiguing exercise protocol for the measurements). Subjects were instructed to perform 2 training sessions per day of 2 min duration each (2 min in the morning and 2 min in the afternoon). They were asked to write a personal training diary, and to note down the time and duration of their training sessions. The subjects were admonished to train by daily messages, phone calls, e-mails, or personal visits. Before the second examination, a photographic image of the ADM of the trained and the untrained hand was taken to assess visually the muscle volume of the hypothenar eminence.

### 2.8 Statistical analysis

All measurement results were expressed in percentage of pre-exercise levels (pre-exercise force and pre-exercise CCI = 100%). Many of the measured parameters were not distributed in a Gaussian way. Hence, non-parametric tests (Wilcoxon signed rank test) were applied throughout to test differences of means. The effect size and the statistical power were calculated using the product moment *r* for the Wilcoxon signed rank test *a posteriori* using $r=|\frac{z}{\sqrt{n}}|$ , where *z* = z-value, and *n* = sample size. Threshold values of *r* for small, medium, and large effect sizes were 0.10, 0.30, and 0.50, respectively. Sample size was calculated also *a posterior* for the largest effect separately using Cohen’s d. Regression analysis (method of least squares) was applied where appropriate. Statistical analysis were performed using the statistical language R version 4.1.2 (R Foundation, Vienna, Austria).

## 3 Results

All subjects tolerated the experiments and the training well without any adverse event. All subjects completed all measurements, both before and after training. Cooperation during the training period was excellent, as judged by the diaries and personal communication. To account for the individual availability of the subjects for pre- and post-training measurements, we allowed some flexibility of the total duration of training. Thus, the duration of training ranged from 14 to 26 days (mean duration: 20.5 days, SD 3.9). Regression analysis revealed no statistically significant influence of training duration on training effects (in particular, on the increases of force and of CCI at the end of the 2 min exercise test).

### 3.1 Force measurements during exercise

Subjectively, the training induced increases of muscle force for little finger abduction in all subjects. A visible hypertrophy of the hypothenar muscles could be seen in most subjects. Due to software restrictions, it was however not possible to measure the force of little finger abduction in absolute units. Reliable force recording of distinct small hand muscles during a fatiguing task was previously shown to be difficult (Merton, 1954; Bigland-Ritchie et al., 1982; Di Giulio et al., 2006). Therefore, in this study, the training induced gain of muscle force could not be quantified.

**FIGURE 2:**
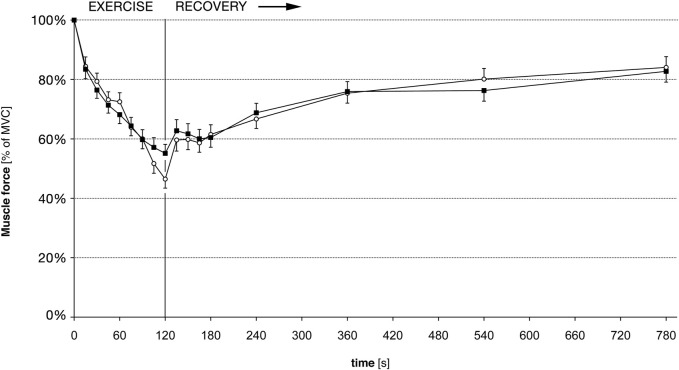
Muscle force for 5th finger abduction, before and after training. Force is given in percent of the maximal voluntary force (MVC); hence a training induced gain of force is not accounted for. White circles: force before training. Black squares: Force after training. Error bars denote standard error of the mean. There are no statistically significant differences of force pre-to post-training.

Nevertheless, our force recordings were well suited to follow the fatigue induced loss of force during the exercise measurements. In all subjects, before and after training, force declined significantly during the 2 min of exercise. There were only small inter-individual differences in the amount of muscular fatigue. Before training, force reached an average level of 46% (SD 11.8%) of MVC at the end of the exercise. After the training, force dropped to 55% (SD 19.8%). This difference of force reduction was not statistical significant compared to pre-training (Figure 2).

### 3.2 Triple stimulation technique amplitude-ratio and CCI during exercise

Before training, the average TST amplitude ratio was 94.5% (SD 17.05%) at the beginning of the exercise. After training, the average TST amplitude ratio was 94.7% (SD 13.14%). Thus, before and after training nearly all target MNs could be brought to discharge in the rested muscle at the beginning of the exercise. Before training, the average CCI was 93.4% (SD 22.64%) at the beginning of the exercise. After training, the average CCI was 94.7% (SD 16.96%) at the beginning of the exercise. The difference between average TST amplitude ratio and CCI was statistically not significant. This was also true at the end of the recovery period, before and after training. Thus, the ratio between TST amplitude ratio and CCI was not affected during our exercise protocol and recovery, and it was not altered by the training. Therefore, the CCI was judged an acceptable replacement for the TST amplitude ratio, as previously shown (Rösler et al., 2009). The small systematical error introduced by the CCI was further accounted for by normalizing data to the pre-exercise values.

**FIGURE 3:**
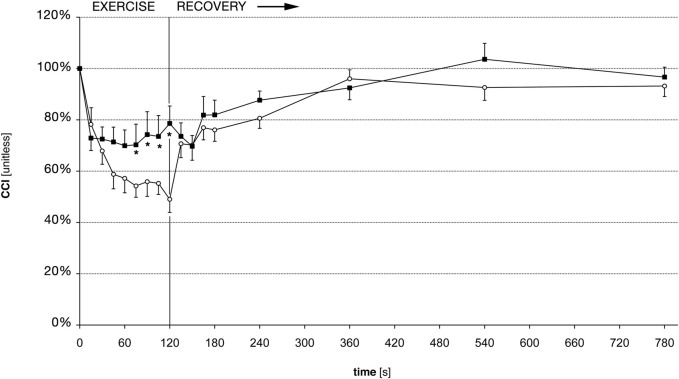
Central conduction index (CCI) before and after training. White circles: CCI before training, black squares: CCI after training. Error bars denote standard error of the mean. * denote statistically significant differences of CCI pre-to post-training (Wilcoxon signed rank test, *p* < 0.05).

**FIGURE 4:**
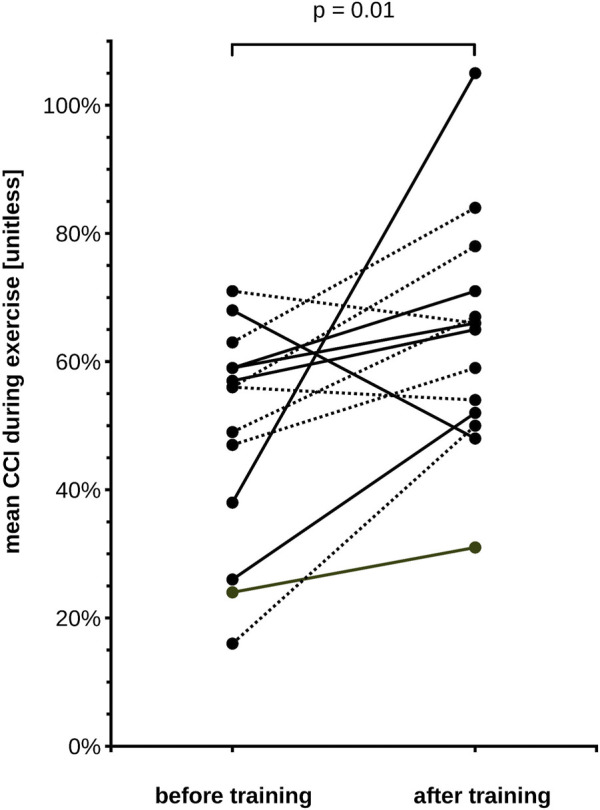
Central conduction index (CCI) during exercise for each of our 15 subjects, before and after training. CCI is averaged across the individual measurements during the 2^nd^ minute of exercise (75–120 s). Note that the mean exercise-CCI increased in 12 subjects and decreased slightly in 3 subjects. The group means differ significantly (Wilcoxon signed rank test, *p*-value in the figure). Solid lines depict male subjects, dotted lines female subjects.

On average, the CCI decreased markedly during the 2 min of exercise. There were important inter-individual differences in the CCI reduction, as seen in an earlier study (Rösler et al., 2009). Before training, the CCI decreased to 49% (SD 23.7%), on average (Figure 3). After the training, the reduction of the CCI was much less marked compared to the experiment before training, since it reached a minimum of 70% (SD 19.8%) at t = 60 s, and 79% (SD 26.4%) at t = 120 s (Figure 3). The pre-to post-training difference was statistically significant as shown in Figure 3 and Table 1. The required sample size calculated *a posteriori* to demonstrate this effect was 15. The average exercise-CCI (i.e., individually averaged CCIs from t = 75 s to t = 120 s) increased significantly with training (Figure 4). Thus, while the training did not change development of muscular fatigue, it significantly reduced CCI drop after 2 min exercise.

**TABLE 1:** Statistical analysis of differences between the time course of CCI recordings before and after training For each time point during exercise and the recovery period, *p*-values, the effect size *r*, and the statistical power are reported, based on Wilcoxon signed rank test. Depending on the value of *r*, effect size is categorized as small (*r* < 0.1), medium (*r* 0.1–0.3), and large (*r* > 0.3).

Time [s]	*p*-value	Effect size (*r*)			Power [%]
		small	medium	large	
15	0.45	0.03			5
30	0.48	0.01			5
45	0.19		0.23		23
60	0.19		0.23		23
75	**0.05**			0.42	66
90	**0.02**			0.53	88
105	**0.02**			0.52	87
120	**0.01**			0.61	96
135	0.66		0.11		9
150	1.00	0.00			0
165	0.32		0.12		10
180	0.21		0.21		20
240	0.15		0.27		31
360	0.38	0.08			7
540	0.39	0.07			7
780	0.61	0.07			7

Bold values emphasize statistically significant results.

### 3.3 Force and CCI during recovery

After the exercise, the CCI recovered faster than the force (Figures 2, 3), as shown previously (Rösler et al., 2009). However, there was no difference in the force recovery before and after training (Figure 2) and there was no difference between the CCI recovery before and after training (Figure 3).

## 4 Discussion

In the present study, we analyzed if the exercise-induced drop of CCI was influenced by muscular training. Our data demonstrate that after approximately 3 weeks of little finger abduction training, the CCI decreased significantly less during exercise. Compared to the experiments in the untrained condition, we found 30% less CCI decrease during 2 min of isometric exercise after muscular training (Figures 3, 4). At the same time, the relative drop of force remained unchanged. During muscular fatigue, TMS fails to activate the entire spinal MN pool of a target muscle. This failure of activation was demonstrated in our study by the drop of the CCI (which is a measure of the size of the motor evoked potential) during a 2 min exercise. Previous studies demonstrated that it was related to the intensity and the duration of exercise, and varied between subjects (Andersen et al., 2003; Rösler et al., 2009). The differential adaptation of force and CCI during training does not support the idea that there is a straightforward causal link between the drop of CCI and the drop of force during motor fatigue.

We used a repetitive contraction protocol, introduced in a previous study (Rösler et al., 2009). Pilot experiments and the study by Andersen et al. (2003) had demonstrated that sustained work principally leads to similar alterations of TMS induced responses as those observed here. The results obtained in the present study before training were virtually identical to those of our previous study (Rösler et al., 2009). Facilitation maneuvers may additionally confound the fatigue induced changes of MN excitability measured by TMS responsiveness, as facilitatory and inhibitory inputs to MNs at both spinal and supraspinal sites may change unpredictably during a fatiguing exercise. To achieve activation of 100% of MNs by TMS, facilitation of the response by voluntary contraction is necessary (Magistris et al., 1998; Rösler et al., 2002). In the present study, we chose contractions of 20% of MVC to facilitate the TST responses throughout the entire exercise protocol. We have previously discussed the implications of the amount of pre-contraction on the facilitatory maneuver for the TST in fatiguing exercise (Rösler et al., 2009).

The technical implications of the TST have extensively been discussed previously, regarding the comparison to conventional MEPs (Magistris et al., 1998; Rösler et al., 1999; Rösler et al., 2002; Andersen et al., 2003; Humm et al., 2004), and to the use of the CCI (Rösler et al., 2009), and the small systematic error introduced due to peripheral desynchronisation and muscle-nerve ephaptic backfiring (Magistris et al., 1998), all not being critical for the interpretation of our data.

During all experiments, effects of peripheral fatigue on the recordings were observed. Peripheral fatigue leads to characteristic changes of the response configuration (broadening of the CMAP, small amplitude reduction). These changes do not influence the CCI, because they occur in both deflections of the TST recordings. As shown previously during a similar exercise protocol, the ratio between the second and first deflection remained constant, if TMS was replaced by peripheral stimulation at the brachial plexus within the TST protocol (Rösler et al., 2009). The observed changes of CCI with TMS are therefore caused by excitability changes within the central motor pathway, and are not influenced by peripheral effects of fatigue.

Our data shows that changes in MEPs (as measured by the CCI) are associated with the loss of muscular force during exercise. During fatigue, fewer MNs are elicited after TMS, suggesting that MNs become less responsive to synaptic input (Taylor and Gandevia, 2008). The present results demonstrate that this phenomenon is influenced by training. After training, our subjects’ CCI decreased significantly less than before training, indicating that the responsiveness to synaptic input during fatigue improved (Figures 3, 4). Previous studies using TMS in high resistance training analyzed the size of MEPs before and after training, and found unchanged or reduced MEP size, depending on facilitatory maneuver and examined muscle (Carroll et al., 2002; Jensen et al., 2005). Our present results show that the lack of a training effect on MEP size may only be observed if measurements are performed in a rested (i.e., unfatigued) muscle, but that differences may be observed if measurements are done during a fatiguing exercise. Indeed, the present measurements showed a CCI near 100% at the beginning of the exercise, before and after training. Hence, nearly 100% of the target muscle MN pool could be brought to discharge in the rested muscle, and this percentage was not affected by the training. That “central neural drive” increases with strength training has previously inferred by observations of surface EMG recordings, which indicates that greater number of motor units can be recruited voluntarily (Grimby et al., 1981). Increased ability of the CNS to activate motor units after strength is also demonstrated by the twitch interpolation technique. During strength training, the maximal voluntary force increases more than the stimulus evoked force, suggesting that a greater proportion of the muscle can be accessed by the CNS after training (McDonagh et al., 1983; Davies et al., 1985; Narici et al., 1989). It is well conceivable that the effect of our training program on the CCI during fatigue is related to the increased central neural drive as described in the above mentioned studies. A limitation of our study is the lack of a control group who underwent no or “sham” training to rule out that our findings are simply an effect of central learning mechanisms. However, previous studies have not found significant differences in motor cortex excitability between strength and skill training (Leung et al., 2015). Furthermore, our data do not pinpoint the exact underlying physiological mechanisms responsible for the less pronounced decrease of CCI during exercise after training. Changes of different neural structures at spinal and supraspinal sites could be involved (spinal MNs, cortico-spinal neurons, subcortical neurons contacted by corticospinal tract fibers and projecting to the spinal MNs, intracortical inhibitory and excitatory interneurons). Possible underlying mechanisms will be discussed subsequently.

From previous studies, there is evidence that resistance training does not lead to changes in the representational organization of the cortex. Adaptations in the motor cortex of monkeys are not induced by repetitive execution of a simple movement (Plautz et al., 2000). In rats, the training-induced reorganization of the movement representation within the motor cortex remains similar, whether the rats performed movement against a low or a high load (Remple et al., 2001). In humans, Carroll et al., 2002 demonstrated that the MEP size decreased after resistance training (but not after a “sham” training of similar movements without resistance), and that this decrease occurred equally if MEPs were elicited by transcranial magnetic stimulation or transcranial electrical stimulation. As electrical stimulation targets the corticospinal neurons directly on the axon distal to the axon hillock, and magnetic stimulation excites the corticospinal neuron trans-synaptically *via* intracortical circuits (Edgley, 1997), the results of Carroll et al. point to changes of functional properties at spinal sites rather than structural changes in the motor cortex. Finally, Z’Graggen et al. (2008) did not find an effect of resistance training on the TMS resting motor threshold. Summarized, these studies argue against the explanation of our results by structural cortical adaptations.

On the other hand, it is equally unlikely that afferent input from the periphery could explain our findings. A reflex inhibition of alpha motor neurons by group III and IV muscle afferents was shown to influence firing frequency of motor neurons during fatigue (Bigland-Ritchie et al., 1986; Woods et al., 1987; Garland and McComas, 1990; Duchateau and Hainaut, 1993), and may change with strength training (Aagaard et al., 2000; Kamen and Knight, 2004; Duchateau et al., 2006). Changing afferent negative feedback could thus influence the size of TMS-evoked responses during training. However, previous studies have shown that afferent negative feedback is probably not an important factor for the exercise induced drop of CCI. Rösler et al. induced muscular contractions by stimulating the ulnar nerve by stimulus trains of 20 Hz, simulating the voluntary exercise protocol as closely as possible (Rösler et al., 2009). While this “imposed” exercise led to similar fatigue of muscle force than voluntary exercise, the CCI remained almost constant throughout the 2 min of exercise, excluding an important contribution of muscle afferents to the CCI. Moreover, after voluntary exercise, the recovery of CCI was not delayed by hemostasis induced by putting a cuff around the upper arm; and recovery of conventional MEP amplitudes after sustained post-exercise muscle ischemia was not disturbed (Taylor et al., 2000). Taken together, it seems unlikely that afferent feed-back was involved in the exercise-induced drop of CCI, and thus it seems not probable as well that the observed training effect was caused by alterations of this feed-back mechanism. However, all previously mentioned studies on afferent feed-back mechanisms were not performed after a training period as our present study.

The less pronounced drop in CCI after training could be caused by a training induced reduction of intracortical inhibition during exercise (Rösler et al., 2009). Impaired intracortical inhibition has been a potential explanation for the reduced CCI decrease in patients suffering from multiple sclerosis (Scheidegger et al., 2012). In these patients intracortical inhibition is probably impaired by a cortical maladaptive process due to structural alterations in motor areas. In our healthy subjects after training, intracortical inhibition might be reduced as a transient physiological response to facilitate the motor task. Accordingly, short-latency intracortical inhibition was previously shown to decrease after 3 weeks of strength training in healthy subjects (Goodwill et al., 2012), and silent period duration was also shown to be reduced after 4 weeks of strength training (Kidgell and Pearce, 2010). Intracortical inhibition could be influenced by the level of voluntary effort perceived during the exercise, in healthy subjects (Rösler et al., 2009), and in patients with multiple sclerosis (Scheidegger et al., 2012) and amyotrophic lateral sclerosis (Vucic et al., 2011). Also, a recent systematic review on the effect of resistance training on human cortical excitability concluded that there is evidence favoring the reduction of intracortical inhibition in motor cortex, though pointing at a high degree of inconsistencies in the studies which were evaluated (Colomer-Poveda et al., 2019). In the present study we did not formally assess the level of perceived exertion of our subjects, but most of them reported a greater ease in performing the exercise task after the training.

In conclusion, 3 weeks of regular muscular training significantly influences fatigue induced drop of motor evoked potentials during an isometric exercise. This may be due to a greater responsiveness to synaptic input during fatigue and hence a greater proportion of muscle that can be activated by the CNS after regular muscle training.

## Data Availability

Upon request, the raw data supporting the conclusion of this article will be made available by the authors, without undue reservation.
